# The effectiveness, equity and explainability of health service resource allocation—with applications in kidney transplantation & family planning

**DOI:** 10.3389/frhs.2025.1545864

**Published:** 2025-05-15

**Authors:** Joris van de Klundert, Harwin de Vries, Francisco Pérez-Galarce, Nieves Valdes, Felipe Simon

**Affiliations:** ^1^ Escuela de Negocios, Universidad Adolfo Ibáñez, Santiago de Chile, Chile; ^2^ Rotterdam School of Management, Erasmus University Rotterdam, Rotterdam, Netherlands; ^3^ Department of Computer Science, School of Engineering, Pontificia Universidad Católica de Chile, Santiago, Chile; ^4^ College of Science and Engineering, University of Minnesota, Minneapolis, MN, United States

**Keywords:** explainability, equity, effectiveness, kidney allocation, family planning, healthcare analytics, explainable AI

## Abstract

**Introduction:**

Halfway to the deadline of the 2030 agenda, humankind continues to face long-standing yet urgent policy and management challenges to address resource shortages and deliver on Sustainable Development Goal 3; health and well-being for all at all ages. More than half of the global population lacks access to essential health services. Additional resources are required and need to be allocated effectively and equitably. Resource allocation models, however, have struggled to accurately predict effects and to present optimal allocations, thus hampering effectiveness and equity improvement. The current advances in machine learning present opportunities to better predict allocation effects and to prescribe solutions that better balance effectiveness and equity. The most advanced of these models tend to be “black box” models that lack explainability. This lack of explainability is problematic as it can clash with professional values and hide biases that negatively impact effectiveness and equity.

**Methods:**

Through a novel theoretical framework and two diverse case studies, this manuscript explores the trade-offs between effectiveness, equity, and explainability. The case studies consider family planning in a low income country and kidney allocation in a high income country.

**Results:**

Both case studies find that the least explainable models hardly offer improvements in effectiveness and equity over explainable alternatives.

**Discussion:**

As this may more widely apply to health resource allocation decisions, explainable analytics, which are more likely to be trusted and used, might better enable progress towards SDG3 for now. Future research on explainability, also in relation to equity and fairness of allocation policies, can help deliver on the promise of advanced predictive and prescriptive analytics.

## 1 Introduction

Halfway to the deadline of the 2030 agenda, humankind continues to face long standing yet urgent policy and management challenges to address resource shortages and deliver on SDG 3; health and well-being for all at all ages (1, 2). More than half of the global population, among whom a variety of subpopulations in high income countries, lack access to essential health services (3, 4). The scarcity of financial, human, and other resources complicates progress towards the “bold commitment” of SDG3 and additional investments are needed to achieve it (1, 5, 6). Without a significant additional investment in health service resources, more than a third of the global population will still lack access to essential health services by 2030 (4).

Traditionally, overall population health has been an important criterion to guide health policy and management decisions on the allocation of financial resources and others. Population health has been operationalized through measures to assess the health-adjusted life years (HALYs) enjoyed by a population, such as the disability-adjusted life years (DALYs) and the quality-adjusted life years [QALYs; (7, 8)]. Policy efforts targetting to maximize HALYs for the population at large may, however, negatively impact the health of some individuals and subpopulations when constrained by resource scarcity. Budget limitations may, for instance, direct policy preferences towards resource allocations that disadvantage subpopulations for which the expected resource effectiveness is lower (9, 10). More generally, resource scarcity and subsequent allocation decisions can easily cause and aggravate differences in health service access and resulting health outcomes. From the inclusiveness perspective of SDG3, which targets health *for all*, this raises the interest in the fairness of health resource allocation decisions and in the avoidance of resulting health inequalities.

The avoidance of health inequalities whenever possible is explicitly considered in the definition of health equity (11). Health equity refers to a fair and just opportunity for all to be as healthy as possible and this definition classifies avoidable inequalities as inequitable (11, 12). Policy decisions regarding the allocation of scarce health services resources can promote health equity by improving equity in access, utilization, and quality of health services, and in the resulting health outcomes (10, 12).

In pursuit of resource allocation decisions that optimally balance the expected resulting effectiveness and equity, scientists and practitioners have been confronted with challenging prediction problems to estimate future health effects of possible resource allocations and subsequent prescription problems to identify the best resource allocation. These challenges regard both the specification of the models and the methods to estimate and solve these models. In the remainder we, somewhat formally, refer to the systematic computational analysis of data by combining mathematical models with corresponding solution methods as *analytics* and focus on the use of analytics in support of health resource allocation decisions (13). Moreover, *predictive analytics* will refer to data-driven models and methods for the purpose of prediction such as the prediction of changes in health outcomes that result from health resource allocation decisions. Likewise, *prescriptive analytics* refers to data-driven models and corresponding methods to solve optimization problems, such as the problem of allocating health resources to obtain the most equitable health outcomes.

Predictive analytics often precede prescriptive analytics in approaches that seek to optimize health service resource allocation. The predictive analytics estimate effects of allocation decisions and the prescriptive analytics uses these estimation and maximizes the expected effects. In various domains, however, the ability of predictive analytics to predict health outcomes has long been modest and has triggered questions whether predictive models provide a valid basis for prescriptive analytics to allocate scarce health service resources [see, for instance (14, 15), within the realm of donor organ allocation]. In recent decades, many researchers have therefore sought to extend the traditional analytics toolkit by exploring artificially intelligence (AI) techniques, in particular machine learning (ML) (16, 17).

These recent advances have brought progress as well as renewed challenges to balancing effectiveness and equity (15, 18). Novel predictive analytics approaches using ML are more likely to be biased and their prediction accuracy may vary across subpopulations, often disadvantaging smaller (minority) subpopulations (18–20). Resource allocation decisions based on biased predictions can subsequently fail to deliver the expected effectiveness and (unintentionally) diminish health equity (15, 18, 21).

These possible drawbacks are perceived as particularly undesirable when using “black-box” or “closed-box” approaches from the analytics toolkit of which the working, the results, or both may be difficult to explain. Such black-box models are particularly prone to produce results that yield intended effectiveness improvements together with unintended and even unobserved inequity increases, or vice versa, while inexplicably violating agreed equity principles and regulations. The criticality of health and equitable access to health services has rendered it an explicit priority area of explainable AI (XAI) (22, 23).

**Figure 1:**
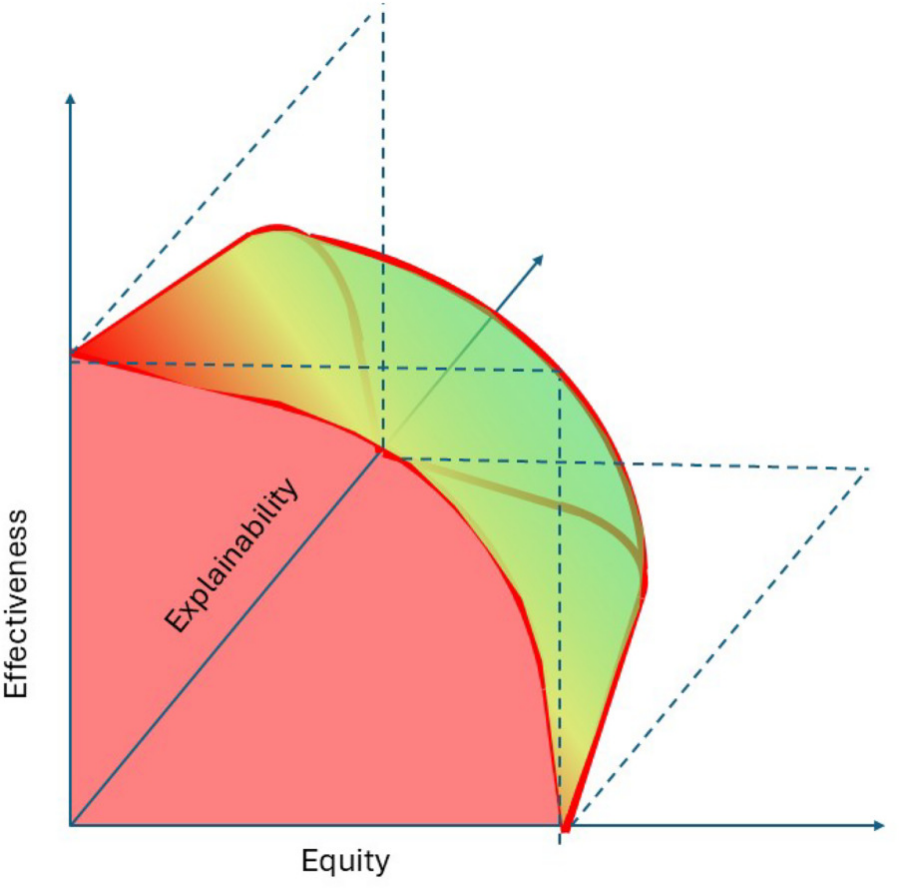
A trade-offs perspective on effectiveness, equity, and explainability.

The emerging literature on XAI in health services mostly consists of case study applications and lacks embedding in a commonly adopted assessment framework (24, 25). Theoretical advances mostly operationalize explainability-related constructs such as fairness and biases from theoretical machine learning and statistics perspectives without developing the relationships with health services measures such as effectiveness and equity. With a view towards achieving SDG3, this research aims to advance the understanding of the explainability of analytics for health resource allocation and of the corresponding interactions between effectiveness, equity, and explainability. We hypothesize that analytics approaches to optimally allocate health service resources harbor trade-offs between these three constructs, as also reflected in Figure 1. The figure reflects that the optimal analytics approaches to resource allocations are situated on the exterior of a three-dimensional performance space in which predictive and predictive analytics can operate. The two-dimensional red front surface represents the effectiveness-equity combinations attainable by non explainable methods. It may be noted that the visualization reflects that the effectiveness-equity plane shrinks as more and more explainability is demanded from prescriptive analytics.

Our quest into these trade-offs will be based on a newly proposed framework which connects effectiveness, equity and explainability to resource allocation decisions. The framework can serve as an instrument to strengthen the contributions of explainable analytics towards achieving SDG3.

We illustrate the framework with two case studies. The selected case studies both present resource allocation problems for large populations and both address major health conditions (26–28). Moreover, they are from domains in which advanced analytics are already making contributions and in which explainability is a key concern (15, 27, 29–32). The first case study is from a low income setting and relates to SDG 3.7 on sexual and reproductive health. It regards the highly prioritized health services for family planning (FP) in low- and middle-income countries (LMIC) (2). The second case study, by contrast, draws from a high income setting and considers the allocation of scarcely available donor kidneys to patients suffering from end-stage renal disease (ESRD). Focusing on this non-communicable condition, it connects to SDG 3.4 which seeks to reduce premature mortality from non-communicable diseases through prevention and treatment by one third. The differences between the case studies provide a form of triangulation that may promote the general validity of the results and interpretations in the context of the framework. Together, the framework and case study results thus provide a basis to reflect on the relationship between effectiveness, equity, and explainability. The discussion section therefore combines the conceptual theoretical perspectives provided by the framework with the practical perspectives offered by the case studies.

## 2 Theoretical framework, materials, and methods

**Figure 2:**
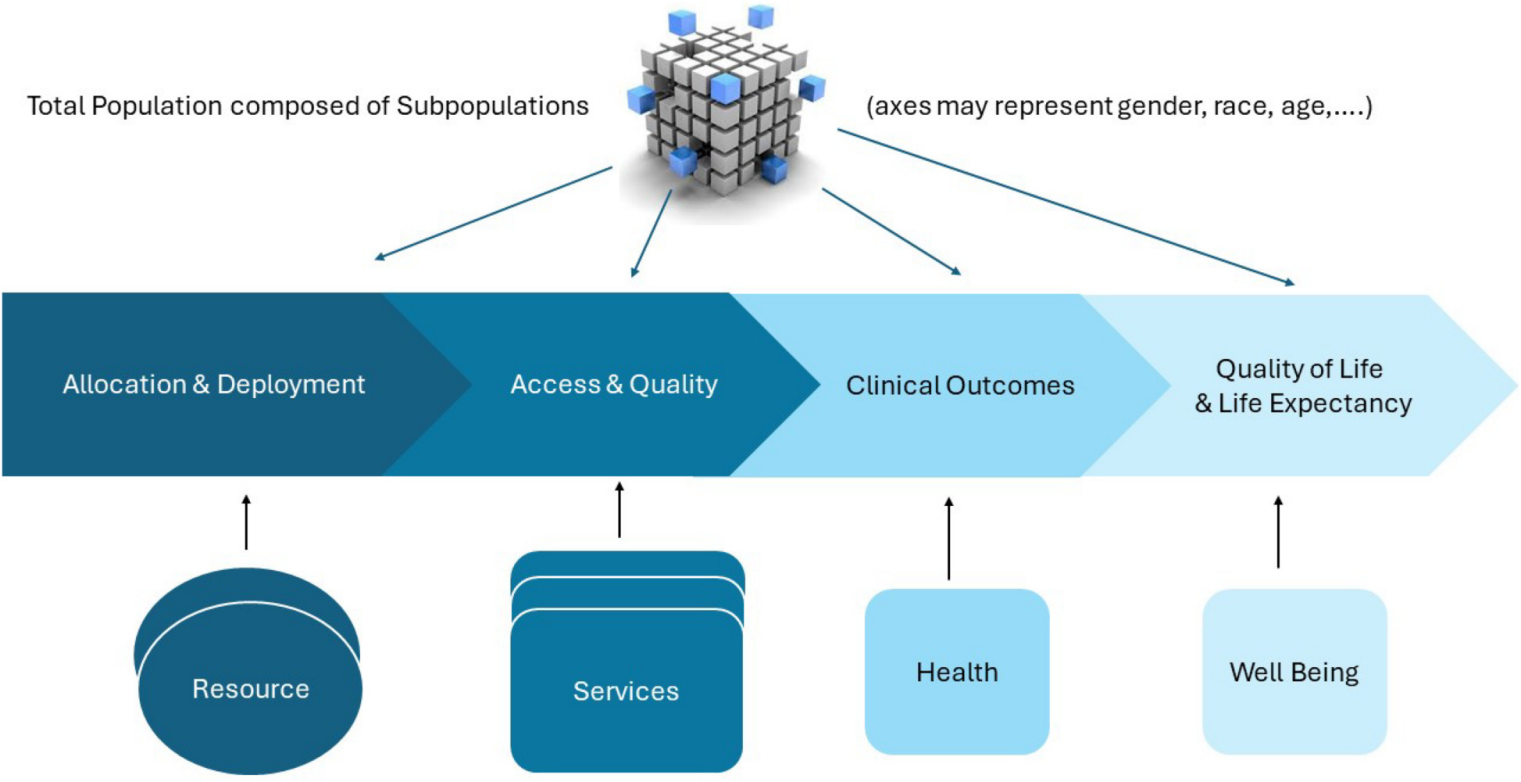
An operational model for resource effectiveness and equity.

The proposed framework, as visualized in Figure 2, is rooted in Donabedian’s well known input-process-outcome model and more contemporary and extensive operational frameworks (33, 34). Through the lens of Donabedian, health services resources to be allocated are part of the health services structure and the health services to be accessed are the processes. The structure and services together determine the outcomes.

On the left, Figure 2 presents the resources, which might be human resources of various degrees of training and disciplines, medical equipment for diagnosis and treatment, drugs, facilities, and financial resources.

Below, we elaborate the framework with a focus on the effectiveness, equity, and explainability of the resource allocation decisions. Thus, the framework requires metrics to express effects of allocation decisions on access to healthcare services and subsequently on the health outcomes achieved as a result of these services (35, 36). The focus on equity and explainability implies an interest in the effects obtained for relevant patient subpopulations as visualized in the top part of Figure 2.

For any set of health services depicted in the bottom part of Figure 2, the cube, or dice, in the top part of Figure 2 represents the subpopulations needing these services. These subpopulations are defined by distinguishing dimensions that are relevant for equity, such as disease severity, age, gender, disability, socioeconomic status, geographical location, et cetera (7). The cube can only visualize subpopulations defined along three dimensions but higher-dimensional definitions can be considered. For example, an equity analysis may distinguish subpopulations along the dimensions age (0–18, 18–65, 65+), gender (M/F), insurance status (public, private) and blood type (O, A, B, AB), potentially defining 3×2×2×4=48 subpopulations.

Following the framework, predictive analytics takes allocation decisions and sometimes subsequent model components as input. Its output is a prediction of “downstream” effects, e.g., in terms of access, quality, health, and well-being. For instance, a straightforward predictive model may estimate effects of resource allocation decisions on service access. A more complex model might take resource allocation decisions and service access and quality measures as predictors and estimate the resulting health and well-being outcomes. Incorporating the results of predictive analytics, prescriptive analytics can identify resource allocation decisions so as to maximize effectiveness, equity, or a combination of both. The prescriptive analytics used may again be relatively straightforward, for instance calculating the increase in number of visits of mobile teams offering FP service that results from enlarging the number of mobile teams. Alternatively, the prescriptive analytics might be more ambitious and aim to establish a set of routes for the mobile teams that maximizes equity in QALYs across the villages populations within a province.

The next three subsections present general measures for the effects of health resource allocation to guide analytics and decision making. The effectiveness subsection operationalizes measures for the total (e.g., sum) of these effects, whereas the equity subsection delves into differences in effects among subpopulations. The third subsection covers the explainability of the working and results of analytics in terms of the effects of the health resource allocation decisions they aim to optimize.

### 2.1 Effectiveness

In Figure 2, access refers to “timely use of services according to need” rather than to alternative definitions which might, for instance, address availaibility or proximity of services (37). Accordingly, the main access dimensions considered are therefore timeliness of services provided and the conformance to need, for which the main effectiveness measures are waiting time and the fraction of patients in need of a service that actually receive it. These measures and more case-specific variants are elaborated in the two case studies.

The World Health Organisation defines the quality of health services as “the degree to which health services for individuals and populations increase the likelihood of desired health outcomes” (38). This definition explicitly defines that health service access may need to vary across subpopulations and individuals according to differences in effectiveness and (patient) values. At the same time, this definition positions quality in relation to the adoption of evidence-based standards that promote effectiveness, i.e., the likelihood of desired outcomes. A second pair of main measures for health service processes thus regard the fraction of services provided in compliance with evidence-based standards and the fraction of patients for which services have been delivered according to their values.

Figure 2 subsequently and relatively narrowly defines health in terms of clinical health service outcomes. It uses health to refer to the direct, clinical, treatment outcomes. Some relevant clinical outcomes may differ per condition, while others such as mortality rate or pain are more generic. Both case studies illustrate effects of resource allocation on specific and generic clinical outcomes.

Health outcomes research importantly focuses on generic metrics regarding health-related quality of life and well-being, as this enables to address effectiveness and equity in a broadly applicable framework and, therefore, also to address resource allocation decisions for a broad set of conditions (39, 40). In Figure 2, these shared outcomes are labeled as “Well-Being” and combine health adjusted quality of life with longevity. We use health adjusted quality of life as a general term encompassing well-elaborated frameworks such as the burden of disease framework, which defines DALYs, and the health-related quality of life (QoL) framework with its QALYs (41, 42). Both case studies present illustrations of the relationship between clinical outcomes and well-being outcomes.

As quality of life typically decreases with age and disability, it is important to distinguish the effectiveness measures life expectancy (LE) and health adjusted life expectancy (HALE). This has caused commonly accepted effectiveness measures to consider QALYs gained or DALYs averted rather than life years gained or lost. As an alternative to these absolute effectiveness measures, one may consider relative increases in (HA)LE (7). For instance, let us suppose that a scarce donor organ can be allocated either to a 30-year or to a 55-years-old patient for a life-saving transplantation. Furthermore, suppose that a successful transplantation might restores their (HA)LE to their original value of 80 years. Then, the relative effectiveness can be viewed to be equal whereas the absolute efectiveness is 50 years for the 30-years-old and only 25 years for the 55-years-old. If the transplantation prolongs life expectancy by 25 years for both patients, the effectiveness is the same in absolute terms, but the 30-years-old will live only a fraction $\left(\frac{30+25}{80}=0.69\right)$ of her original (HA)LE whereas the 55-years-old returns to the original (HA)LE.

Clearly, such differences in effects on outcome measures not only impact effectiveness, but also impact equity and explainability, as discussed in the next subsections.

### 2.2 Equity

Equity measures can be defined in association with any of the aforementioned effectiveness measures. However, while effectiveness considers the sum of the outcomes obtained for selected subpopulations, equity is primarily defined on the basis of differences in effects between subpopulations.

As mentioned in the introduction, not every inequality in health service access, quality of services, health outcomes, or well-being outcomes implies inequity. Inequalities are inequitable in so far as they entail avoidable, unfair, or unjust disparity in opportunity for all to be as healthy as possible (43, 44). For example, genetic variety can cause differences in health outcomes resulting from resource allocation decisions that are beyond the scope of the decision space. Likewise, limitations in the resources to be allocated may imply that not all subpopulations can be fully serviced according to need. In such a case, elements of lottery may be considered as fair and equitable allocation mechanisms even if they cannot avoid inequalities in access and outcomes (45, 46).

Inequity in the effects of resource allocation decisions can be expressed through pairwise effect comparison between subpopulations or through the distribution of resource allocation effects over subpopulations. The Gini coefficient is a widely recognized measure that summarizes information about the distribution of an effect, measuring inequality on a scale from 0 to 1 (47). Higher values indicate greater inequality, with 0 representing perfect equality, e.g., in case all population members enjoy the same health outcome. Conversely, the value 1 indicates perfect inequality, where the maximum possible health outcome (e.g., HALE) is achieved for one person (or subpopulation) and the minimum is achieved for everyone else.

**Figure 3:**
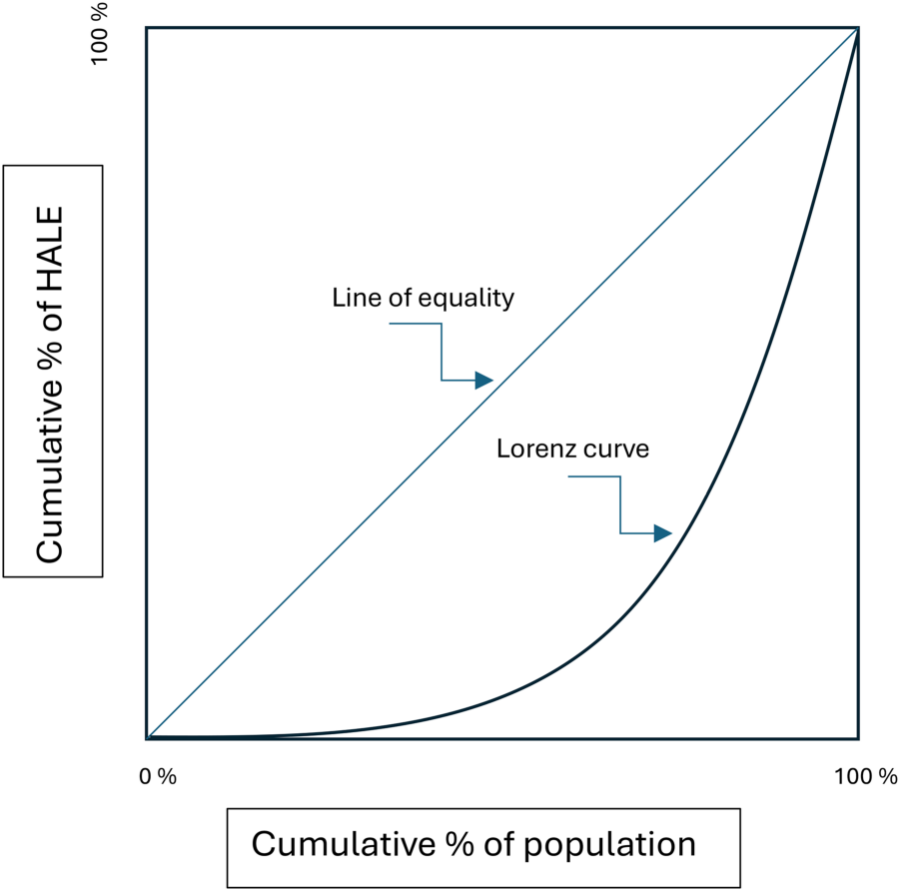
Lorenz curve.

The Gini coefficient is linked to the Lorenz curve, a graphical representation of the distribution of an effect. In the HALE example, the Lorenz curve shows the cumulative distribution of HALE (vertical axis in Figure 3) by successive percentiles of the population (horizontal axis in Figure 3) (48). In case of perfectly equal distribution, the Lorenz curve forms a straight diagonal line, known as the “line of equality.” The Gini coefficient is defined as the surface of the area between the Lorenz curve and this line of equality.

Viewed from the perspective of a Lorenz curve, Rawls’ theory of justice considers allocation decisions equitable if they (recursively) maximize the minimum effectiveness over all subpopulations (49). Thus, it would consider a Gini coefficient of a strictly positive value equitable if (recursively) the contribution to the Gini value by the subpopulation for which effectiveness is lowest cannot be improved (e.g., because of genetic differences).

As Rawls’ definition of distributive justice fully prioritizes maximizing the minimum effects over the subpopulations and disregards consequences for the total effectiveness over all subpopulations, it defines an extreme in the equity-effectiveness trade-off spectrum. It has been applied to design equitable organ allocation policies that eliminate access inequalities among populations depending on blood type and current allocation practices in the US have adopted these principles (31, 50). The other extreme is formed by maximizing effectiveness with complete disregard of differences in effects between subpopulations.

The Atkinson inequality measure (or Atkinson index) facilitates less extreme approaches on the equity vs. effectiveness trade-off spectrum depicted in Figure 1. Like the Gini coefficient, it is associated with a social welfare function, which, in this case, multiplies the average effect across a population with an equity measure (the index) (51). It expresses the effectiveness vs. equity trade-off in terms of the “equally distributed equivalent” level of the effect, defined as the fraction of the total effect that a population would need to sacrifice to achieve a more equitable distribution (52). For example, the corresponding social welfare function for analytics with zero explainability is defined by the (transparent) front facing side of the three dimensional body depicted in Figure 1 and it can be used to identify solutions corresponding to normative choices of Atkinson index values.

### 2.3 Explainability

Explainability of analytics can refer to the working of the methods used and to the results obtained by estimating or solving an analytic model (23, 53). The definitions provided for explainability in this rapidly developing field of science vary and build on definitions of interpretability, understandability, transparency, and comprehensibility, and vice versa (23, 53, 54).

In the remainder, we define *explainability* of analytics as the extent to which the working and solutions provided by analytics can be explained to an audience of relevant human stakeholders (where the explainer can be another human or an AI technology). Following this non-dichotomous definition, the explainability of models ranges across a continuum as is also depicted in Figure 1.

Explainability is particularly important for “critical” sectors that impact human safety and health (23, 53). Consequently, explainability should enable relevant stakeholders to verify and trust that health resource allocation decisions are effective and equitable. Building on (53), potentially relevant human stakeholders for health resource allocation decisions are patients, care givers, medical professionals, managers of health service organisations, insurers and funders, regulators, and professionals responsible for developing and operating the analytics technology.

Differences among these stakeholders entail differences in explainability requirements. It has therefore been argued that stakeholders must work together to harness the benefits that analytics technologies can bring to health care and to foster trust (55). Trust is difficult to establish without transparency, for instance, in the form of disclosing to all stakeholders how decisions impacting access and outcomes are made (56).

For predictive analytics, transparency thus requires full disclosure of the models used, the predictors, the data sets used for model estimation, and the methods by which they are parametrized. For prescriptive analytics, it also requires a description of the optimization models and methods. Transparency can be readily provided for classical techniques such as maximum likelihood estimation in linear regression (predictive) and linear programming (prescriptive). Full disclosure can be far more challenging for advanced machine learning approaches, for instance when using deep learning, that may rely on large numbers of hyperparameters that can in turn be tuned by machine learning methods (57, 58). In such cases, the need for trust and verification of ethical principles, compliance to evidence-based standards, and equity requirements bring about challenges for the explainability of the working and outcomes of the model to all relevant stakeholders. In relation to the presented case studies, for example, explainability might require providing evidence-based arguments to visit some villages more frequently than others for the provisioning of FP services, or to prioritize certain patient populations on the kidney transplant wait list.

A main challenge for all predictive and prescriptive analytics is to avoid biases, as they can negatively and unfairly impact effectiveness and equity. Biases of many forms can easily and unintentionally enter healthcare analytics applications, as already evidenced in various contexts (18, 19, 55). Biases may exist in data sources used, such as electronic health records and data from experiments with biased designs. Such biases can be “learned” by analytics, leading to biased predictions and prescriptions. Prescriptive analytics may, for instance, inequitably allocate fewer resources to subpopulations for which service effectiveness is underestimated. Such biases are particularly likely for minority subpopulations who are naturally under-represented in data sources (a form of data imbalance) and for subpopulations already experiencing access inequities. Biases may also arise as various measures of prediction performance (e.g., calibration measures and discrimination measures) are conflicting (20). Reducing one bias may then enlarge another. These conflicts can also arise in relation to equity measures and among equity measures as illustrated above for kidney allocation.

Transparency and explainability facilitate stakeholders to notice biases and take corrective actions to improve the model or when considering model results in support of resource allocation decisions. Conversely, a lack of transparency and explainability may complicate or even block the implementation and use of analytics when it is perceived to lead to violations of ethical principles or forms of discrimination that are explicitly addressed in guidelines, codes of conduct, regulations, and law (20, 55). Post hoc explainers—forms of artificial intelligence that explain why certain outcomes are obtained—can promote explainability but may have limited value when requesting explanations regarding equity and effectiveness. In fact, such add-on analytics can diminish prediction performance, as has been illustrated for transplant survival prediction (54, 59). As post-hoc explainers may even give “false impressions” of understanding and contribute little to transparency and trust (60), we have not included them in our framework and analysis (but do acknowledge their potential as a future research direction).

Hence, while from a theoretical perspective explainability requirements limit the capabilities of analytics to promote effectiveness and equity, these requirements may promote effectiveness and equity in practice. The latter would contradict the hypothesized trade-off between explainability on the one hand and effectiveness and equity on the other. We use case study as the method to provide further, in-depth, exploration of these trade-offs. As mentioned and motivated in the introduction, we specifically conduct two very diverse case studies in which we apply the framework presented in Figure 2 and adopt predictive and prescriptive analytics of various degrees of explainability for resource allocation. We test our hypotheses regarding trade offs by evaluating the effectiveness and equity achieved for the various degrees of explainability. Correspondingly, the methodological choices within the case studies are covered in the case study subsection rather than in this general methods section.

## 3 Results

### 3.1 Low income case study: family planning

The need for FP services goes unmet for more than 218 million women in LMIC (28). FP services improve health and well-being outcomes as they prevent unintended pregnancies and unsafe abortions, significantly reduce infant and maternal mortality, and strongly benefit economic growth (61). Mobile outreach teams play an important role in scaling up access to FP services in underserved areas such as remote rural areas. These teams visit communities with regular time intervals to offer FP services for free or at low cost.

Each team typically serves a fixed set of sites (i.e., communities) where access to alternative FP providers is low (32). A site visit usually lasts one day, during which the team travels to the site, provides FP services (distributing, dispensing, providing, removing, and counseling on contraceptive methods), and returns home. Teams commonly operate around 220 days per year (30). As such, each team faces a *resource allocation problem*: It must choose how many days (visits) per year to allocate to each outreach site (29). Solving this resource allocation problem using prescriptive analytics involves addressing the trade-offs between effectiveness and equity, as estimated with predictive analytics (29, 30, 32). As outlined below, the rural low-income setting brings about specific explainability needs.

The number of days allocated to a site determines the time interval between consecutive visits and therefore strongly affects *access* to FP services for the site. Using longitudinal data from outreach teams from three African countries and predictive analytics, (30) estimate that visiting a site once per six months instead of once per month reduces the yearly number of FP client visits from that site by 73%–82%. Increasing the number of outreach visits to a given site thus increases the number of FP client visits, which subsequently enhances both contraceptive prevalence and the protection from unwanted pregnancy (62). The latter is not only an important outcome in itself, but is also associated with strong improvements in outcomes such as maternal and infant mortality (61). Satisfying the unmet need for FP might avert 104,000 maternal deaths per year (63).

Allocating equal numbers of outreach visits to each site appears to offer equitable access, yet it fails to recognize differences in access needs among sites. The guidelines and outreach programs therefore typically recommend higher visit frequencies for sites with higher needs and demand (30, 64). The guidelines thus reflect one of the two key objectives of FP outreach programs: to maximize *effectiveness*. Two commonly used effectiveness measures are: (1) the number of client visits and (2) the number of couple-years of protection (CYPs) (32). The second objective provides an *equity* perspective: subpopulations whose outcomes are more negatively impacted by lack of access need more resources (mobile team days) allocated to obtain the equal health outcomes. For FP services, this especially applies to young clients and families who experience difficulties accessing FP services through alternative channels (e.g., due to poverty or distance) (61).

FP outreach programs thus face the problem of optimizing the number of visits to allocate to each outreach site with respect to effectiveness (total number of client visits or CYPs) and equity (relative number of client visits or CYPs to subpopulations of young clients and of clients who have difficulties accessing FP services elsewhere). The access measure client visits is strongly and linearly correlated with the clinical outcome measures (32) and can therefore serve as the effectiveness measure. Following an Atkinson-based equity weighting approach, effectiveness and equity objectives can now be combined by weighting visits to the subpopulations of young clients and the number of people in a site with difficulties to access FP services elsewhere. In the illustration below, a weight of 1.5 is used as a proxy for the larger impact on well-being outcomes.

As (30) show based on data from more than 20,000 outreach visits, current allocation decisions are weakly aligned with the aforementioned objectives and often far from optimal. At the same time, black-box *prescriptive analytics* techniques have been highly successful at solving this type of resource allocation problems (65). They take the characteristics of each team and each site as input and return the visit frequencies that are predicted to yield the highest weighted number of client visits or CYPs.

However, the lack of explainability of black-box prescriptive analytics techniques forms an implementation barrier for several salient FP stakeholders in LMICs (30, 32). First, policy makers and mobile team members lack trust in this form of analytics. Second, the black-box nature is difficult to marry with professional values about evidence-based standards. Explaining the solutions (i.e., visit schedules) returned by black-box techniques is perceived to be hard and compliance with evidence based principles is therefore perceived to be difficult ascertain (66). Third, as with any model, biases lie in wait. To give one example, outreach teams tend to schedule site visits during market days and vaccination campaigns, as these attract many potential clients. If a model lacks this information (which is indeed not systematically collected), it may strongly overestimate the “baseline” number of client visits or CYPs in the corresponding site and recommend a higher-than-optimal visit frequency. The lack of explainability of the solutions provided by prescriptive analytics bears the risk that FP providers fail to correct for the biases underneath.

Explainable methods for choosing visit frequencies may assign villages to a limited number of categories, each with its own visit frequency (e.g., three categories with visits every one, three, or six months, respectively) [see (30)]. Metrics of varying degrees of explainability can be used for this categorization. More explainable metrics rely on simpler models to capture the relationships between visits and outcomes. For example, a simple and explainable approach allocates sites to categories in decreasing order of their average number of clients served per visit.

**Figure 4:**
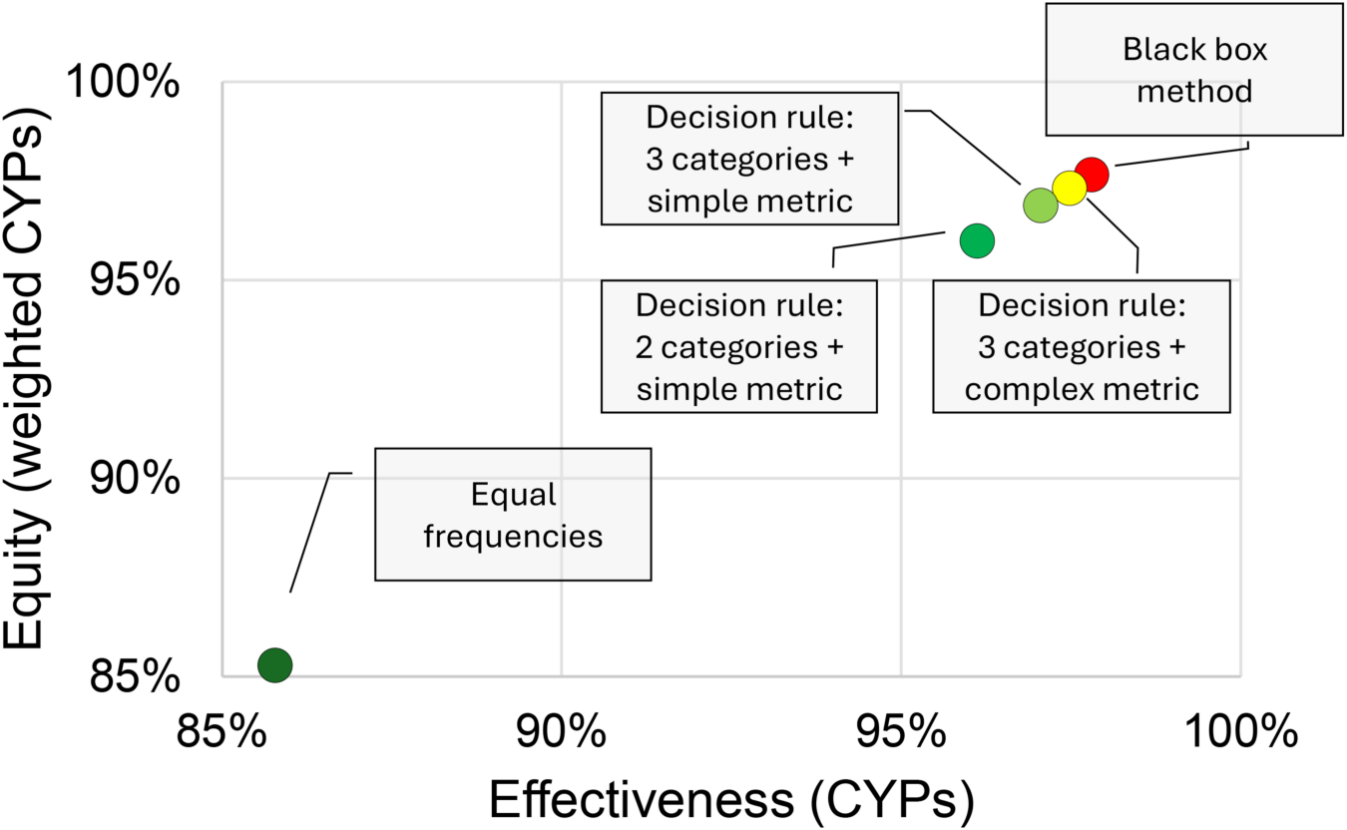
Evaluation of methods for choosing visit frequencies in terms of effectiveness (total CYPs), equity (weighted CYPs), and explainability (green → red = high → low explainability).

Figure 4 summarizes the results of simulation studies to assess the effectiveness and equity achieved by the various approaches, using Uganda as a case study [see (30) for the data]. MATLAB R2019b was the software used for the computational analysis, including the explainable prescriptive analytics and the least explainable exact approach taken from (67).

Figure 4 follows the lay-out of Figure 1 in presenting a two-dimensional graph to visualize the effectiveness-equity trade-off and uses the same color scheme to represent explainability. As expected, the black-box method outperforms the other methods in effectiveness and equity, while the highly explainable method of allocating the same number of outreach visits to each site (“Equal frequencies”) performs poorly. More surprisingly perhaps, the aforementioned simple and explainable approach yields decisions that are less than 1% from optimal with respect to both equity and effectiveness.

Both the method (essentially a decision tree) and the metric used to assign a village to a visit frequency category (the average number of clients or CYPs per outreach visit) can easily be explained. FP providers can therefore easily leverage their local knowledge to identify sites for which this metric is not an accurate metric for categorization (e.g., due to the aforementioned biases) and manually adjust those.

### 3.2 High income case study: kidney allocation in the US

End-stage renal disease (ESRD) is the 12th most common cause of death globally, with a global burden of disease of 35.8 million DALYs in 2017 (68). It is associated with sedentary lifestyle-related risk factors and highly prevalent in high-income countries such as the US. Transplantation is the most cost-effective treatment for ESRD and the number of transplanted kidneys reached a record high of 26,309 in the US in 2022 (69). The number of transplants is, however, limits access to the most effective treatment of transplantation in the US, where almost 90,000 patients are waitlisted for transplantation and one person dies from kidney disease every 9 min (26, 27). Dialysis and transplantation are costly treatments and pose huge strains on the financial resources of health systems. In the US, the annual expenditure for these treatments exceeds 130 billion USD (27). Despite the allocation of these financial resources, kidney disease may still be the chronic disease with the largest inequities in the US (27). For several decades already, these inequities in treatment access and outcomes have been associated with model biases and the explainability of newly developed analytics models (15, 31).

The persistent scarcity of kidneys available for transplantation limits access and many patients remain wait listed for multiple years. Recipients who receive a transplant have a mean waiting time of around three years in 2022 and 12.4% of recipients had been waiting for at least five years (31, 69). In recent years, less than half of the patients enrolled eventually receive a transplant via the wait list (70). Several adjustments in the donor kidney allocation policy have sought to resolve the inequities in this probability of receiving a transplant and in the waiting time until transplantation among subpopulations depending on blood type, age, and ethnicity (69–71).

A first factor the kidney allocation system (KAS) considers for effective and equitable organ allocation is the number of days a patient is on the wait list (72). The expected survival time after transplantation is another important consideration. It depends on patient-related predictors, on donor-related predictors, and on predictors estimating the quality of the match between the donor organ and possible recipients, e.g., in terms of blood type and HLA (72). Predictive analytics for post transplant survival prediction are a rapidly emerging field in which many new machine learning approaches have recently been proposed to improve predictions and allocation decisions (73, 74).

In this case study, we implement a classical Cox proportional hazard model for (death-censored) graft survival prediction, as underlies current KAS parameters (72, 75–77). Cox proportional hazard models assume the graft failure probability is determined by a recipient independent time dependent base line survival function and by a time independent recipient dependent hazard rate (75) and can be estimated using commonly available standard software. Additionally, this case study presents two commonly applied standard machine learning based survival prediction analytics models availaF survival forest model. Survival decision trees and random survival forests are extensions of classical survival trees and random forests for survival prediction with right censored data for which there is evidence of good prediction performance (73). The simplicity and deterministic nature of survival decision trees causes them to be classified as explainable, unlike random survival forests.

The predictive analytics use standard predictors considered in UNOS’ current allocation policy. These include predictors from the kidney donor profile index (KDPI) and from the estimated patient transplant survival score (EPTS) (72, 78, 79). It uses the UNOS/OPTN data fo transplants from the years 2011 to 2013 and corresponding survival data until 2018. The predictive models use single imputation and ten Monte Carlo cross-validation repetitions with an 80%/20% split between training and test data. The predictive models were implemented in Python using scikit-survival. This code and the code for the allocation simulations are available on Github as indicated in the data availability statement below.

We firstly report on predictive analytics for survival prediction. For each of the three prediction models, we present the mean signed prediction error, the Brier score, and the C-index for 5-year post-transplant death-censored donor kidney survival, which is the main clinical (health) outcome of interest (76, 80, 81). These metrics are reported for the ethnicities and genders distinguished by UNOS/OPTN. The subpopulation C-index reported below is novel and calculates the C-index by only considering ordered pairs for which at least one of the recipients belongs to the subpopulation.

In Table 1, green indicates a “positive” bias and red a “negative” bias. There are no noteworthy significant differences in prediction performance among the models, despite their differences in explainability. However, there are significant differences and therefore biases in prediction performance between subpopulations.

**Table 1:** Equity of prediction performance.

Category group	CPH	SDT	RSF
	μ (σ)	μ (σ)	μ (σ)
Calibration			
Brier score			
Overall	0.221 (0.002)	0.224 (0.001)	0.222 (0.001)
Female	0.217 (0.004)	0.219 (0.002)	0.216 (0.003)
Male	0.225 (0.001)	0.228 (0.002)	0.226 (0.002)
Amer Ind/Alaska native	0.256 (0.022)	0.248 (0.021)	0.251 (0.018)
Asian	0.202 (0.011)	0.207 (0.010)	0.200 (0.006)
Black	0.239 (0.006)	0.240 (0.004)	0.238 (0.004)
Hispanic	0.210 (0.003)	0.208 (0.006)	0.204 (0.007)
Multi-racial	0.221 (0.111)	0.237 (0.048)	0.239 (0.048)
Native Hawaiian/Pacific	0.220 (0.019)	0.193 (0.045)	0.192 (0.033)
White	0.215 (0.003)	0.221 (0.002)	0.219 (0.002)
Mean signed error			
Overall	−0.034 (0.011)	−0.033 (0.013)	−0.032 (0.005)
Female	−0.013 (0.015)	−0.016 (0.017)	−0.011 (0.009)
Male	−0.047 (0.010)	−0.044 (0.013)	−0.045 (0.007)
Amer Ind/Alaska native	−0.093 (0.084)	−0.115 (0.047)	−0.117 (0.072)
Asian	0.057 (0.027)	0.065 (0.041)	0.065 (0.021)
Black	−0.068 (0.012)	−0.066 (0.019)	−0.073 (0.01)
Hispanic	0.017 (0.022)	0.022 (0.024)	0.022 (0.027)
Multi-racial	−0.291 (0.321)	−0.209 (0.161)	−0.226 (0.123)
Native Hawaiian/Pacific	0.008 (0.111)	0.057 (0.121)	0.068 (0.102)
White	−0.04 (0.009)	−0.042 (0.014)	−0.034 (0.008)
Discrimination			
C-Index			
Overall	0.610 (0.004)	0.600 (0.004)	0.610 (0.004)
Female	0.582 (0.007)	0.575 (0.008)	0.586 (0.006)
Male	0.627 (0.006)	0.614 (0.006)	0.624 (0.005)
Amer Ind/Alaska native	0.664 (0.055)	0.673 (0.055)	0.649 (0.060)
Asian	0.629 (0.032)	0.613 (0.029)	0.631 (0.025)
Black	0.585 (0.013)	0.564 (0.013)	0.567 (0.008)
Hispanic	0.647 (0.015)	0.645 (0.018)	0.650 (0.019)
Multi-racial	0.615 (0.234)	0.56 (0.166)	0.512 (0.19)
Native Hawaiian/Pacific	0.783 (0.107)	0.812 (0.103)	0.782 (0.099)
White	0.616 (0.007)	0.610 (0.01)	0.629 (0.007)

CPH, cox proportional hazard model; STD, survival decision tree; RSF, random survival forest.

Green indicates a “positive” bias.

Red indicates a “negative” bias.

The Brier score is significantly larger for the Black subpopulation, indicating that their 5-year survival probabilities are estimated less accurately. This can be explained from the (negative) mean signed error, which shows that their survival is significantly overestimated. The mean signed error also reveals that the 5-year survival is significantly underestimated for Asian Americans.

For Hispanics, the Brier score is significantly better than average as is also confirmed by the C-index results, which show that predicting who survives longest is more frequently correct for Hispanics in all three models and less frequently correct for the Black subpopulation for the decision trees and random forests. The C-index scores for some minority subpopulations are even further from average but the difference is not significant, likely because of the small population sizes and resulting large standard error. Lastly, we may note that the female subpopulation score significantly worse on these discrimination measures compared to the majority subpopulation of men.

To verify whether any of the prediction biases results from data imbalance, we have estimated the three prediction models again for a data set in which the large White and Black subpopulations were undersampled to be of the same size as the subpopulation of Hispanics (originally around 15% of the entire recipient population). The prediction results are very comparable as can be verified from the Supplementary Material.

The predictive analytics subsequently feed into prescriptive analytics to allocate organs and impact their effectiveness with equity. This case study considers the effects of allocation on equity in access and on equity in health outcomes among subpopulations according to ethnicity and gender.

The survival predictions are especially relevant for prescriptive analytics models that aim to maximize effectiveness in therms of the health outcome graft failure. An effectiveness maximizing policy allocates organs becoming available to a compatible patient on the wait list with highest expected death censored graft survival resulting from the transplant. In case of ties, the organ may be assigned to the longest waiting patient.

Table 2 presents results obtained using 30 effectiveness maximizing allocation policy simulations for each of the three prediction models. Each simulation spans a period of 30 years, with a warm up period of 8 years. In the allocation policy simulations, compatibility is defined following the blood type compatibility guidelines in KAS and ignores HLA compatibility (72, 78, 79).

**Table 2:** Results obtained by various prediction models in a predictive model that maximizes effectiveness.

	TP		WTUT		ESAT	
Group	μ	σ	μ	σ	μ	σ
CPH						
Total population	0.646	0.018	237	24	2,677	9
Amer Ind/Alaska native, Non-Hispanic	0.718	0.067	210	146	2,621	86
Asian, Non-Hispanic	–0.563	0.037	318	97	2,634	37
Black, Non-Hispanic	–0.688	0.016	–190	22	–2,707	11
Hispanic/Latino	0.624	0.024	305	42	–2,635	19
Multiracial, Non-Hispanic	0.660	0.075	277	226	2,743	119
Native Hawaiian/Other Pacific, Non-H	0.407	0.133	349	614	2,683	173
White, Non-Hispanic	0.630	0.021	242	28	2,674	12
STD						
Total population	0.609	0.019	1,279	77	2,825	19
Amer Ind/Alaska native, Non-Hispanic	0.552	0.051	1,228	88	2,843	226
Asian, Non-Hispanic	0.592	0.035	1,279	80	2,801	128
Black, Non-Hispanic	0.587	0.021	1,275	79	2,833	48
Hispanic/Latino	0.625	0.016	1,224	76	2,881	59
Multiracial, Non-Hispanic	0.637	0.089	1,350	103	2,810	347
Native Hawaiian/Other Pacific, Non-H	0.612	0.136	1,294	107	2,942	404
White, Non-Hispanic	0.623	0.022	1,296	77	2,800	37
RSF						
Total population	0.629	0.019	587	43	2,977	3
Amer Ind/Alaska native, Non-Hispanic	0.435	0.060	794	347	2,970	48
Asian, Non-Hispanic	0.591	0.041	690	111	2,962	21
Black, Non-Hispanic	0.586	0.022	619	59	2,971	6
Hispanic/Latino	0.562	0.026	799	87	2,965	12
Multiracial, Non-Hispanic	0.513	0.081	477	328	2,986	56
Native Hawaiian/Other Pacific, Non-H	0.473	0.113	1,823	1,024	2,966	89
White, Non-Hispanic	–0.700	0.017	–481	41	2,987	7

CPH, cox proportional hazard model; STD, survival decision tree; RSF, random survival forest.

Green indicates a “positive” bias.

Red indicates a “negative” bias.

For the Cox Proportional Hazards model, the results reflect the prediction biases which overestimate survival for the Black people and underestimate survival for Asians. Hence, Black people are more likely to be selected for transplantation and have significantly higher transplant probabilities, while the opposite is the case for Asians. Correspondingly, the Black subpopulation also experiences significantly lower waiting time to transplant and longer expected death censored graft survival after transplant. The latter may not materialize because of the mentioned overestimation.

No significant differences in organ allocation metrics appear among the subpopulations when adopting the survival decision tree predictions. Even more remarkable are the significantly lower overall transplant probability and significantly larger mean waiting time in comparison to the Cox proportional hazards results. The survival decision tree prediction model hardly uses patient related predictors and therefore encounters many ties during allocation. Following the tie-breaking rule, it subsequently assigns the donor organs to the longest waiting among the tied patients. As a result, it very closely mimics the FIFO policy discussed below. This policy can indeed result in equal transplant probabilities and waiting times among ethnicities (31, 50).

The results for the random survival forest based predictions highlight prediction biases that were not apparent from the prediction model results. The predictions associate longer survival with features that are more common in the White subpopulation than in the Hispanic subpopulation and in the Indigenous minority subpopulation. This causes inequity in the form of significant differences in transplant probabilities and transplant waiting times. These differences might reflect existing health and health system biases, such as White patients enrolling at earlier stages of renal disease or having a shorter history of diabetes (50). Altogether, regardless of explainability, embedding ML based prediction models in the resource allocation policies has not led to better allocation performance than embedding classic Cox proportional hazard model.

To counter inequities, a Rawlsian approach to organ allocation may strive for equal transplant probabilities and waiting times (31). A FIFO policy selects the longest waiting compatible patient regardless of expected survival or ethnicity and can therefore achieve equality of transplant probability and waiting time for those who receive a transplant. The choice of survival prediction model therefore becomes irrelevant for FIFO policies. The FIFO allocation results are presented in the Supplementary Material and in Table 3.

**Table 3:** Results obtained by various allocation policies using a Cox proportional hazard prediction model.

	TP		WTUT		ESAT	
Group	μ	σ	μ	σ	μ	σ
Maximize survival effectiveness						
Total population	0.646	0.018	237	24	2,677	9
Amer Ind/Alaska native, Non-Hispanic	0.718	0.067	210	146	2,621	86
Asian, Non-Hispanic	0.563	0.037	318	97	2,634	37
Black, Non-Hispanic	0.688	0.016	190	22	2,707	11
Hispanic/Latino	0.624	0.024	305	42	2,635	19
Multiracial, Non-Hispanic	0.660	0.075	277	226	2,764	119
Native Hawaiian/Other Pacific, Non-H	0.407	0.133	349	614	2,683	173
White, Non-Hispanic	0.630	0.021	242	28	2,674	12
Maximize access equity						
Total population	0.610	0.019	1,253	76	2,579	10
Amer Ind/Alaska native, Non-Hispanic	0.566	0.055	1,155	85	2,640	129
Asian, Non-Hispanic	0.592	0.036	1,276	85	2,561	63
Black, Non-Hispanic	0.587	0.022	1,263	77	2,608	23
Hispanic/Latino	0.626	0.018	1,194	77	2,561	35
Multiracial, Non-Hispanic	0.648	0.088	1,358	142	2,650	188
Native Hawaiian/Other Pacific, Non-H	0.614	0.145	1,276	133	2,487	229
White, Non-Hispanic	0.623	0.022	1,267	78	2,565	21
Hybrid optimization balancing effectiveness and equity						
Total population	0.623	0.019	988	70	2,648	7
Amer Ind/Alaska native, Non-Hispanic	0.550	0.052	707	184	2,643	71
Asian, Non-Hispanic	0.549	0.035	1,231	141	2,630	38
Black, Non-Hispanic	0.644	0.021	835	62	2,671	12
Hispanic/Latino	0.600	0.017	1,056	89	2,631	17
Multiracial, Non-Hispanic	0.674	0.079	903	152	2,615	86
Native Hawaiian/Other Pacific, Non-H	0.548	0.111	1,805	480	2,651	118
White, Non-Hispanic	0.627	0.024	1,064	79	2,638	9

TP, transplant probablity; WTUT, waiting time until transplant; ESAT, expected survival after transplant.

Red indicates a “negative” bias.

Lastly, we consider a hybrid approach which is a hybrid combination of the two aforementioned allocation policies. Whenever an organ becomes available it considers a weighted sum of expected survival and waiting time for each compatible reicipient on the waitlist and selects the recipients with the highest weighted sum. In the results reported in Table 3, the weight for estimated survival is ten times the weight of the waiting time. Current allocation policies also adopt such a weighhted approach (72). As this hybrid approach quickly converges to the FIFO approach when using the survival decision tree and random survival forest models, we present the results for all three policies for the Cox proportional hazard model in Table 3 and refer to the Supplementary Material for the other hybrid results.

**Figure 5:**
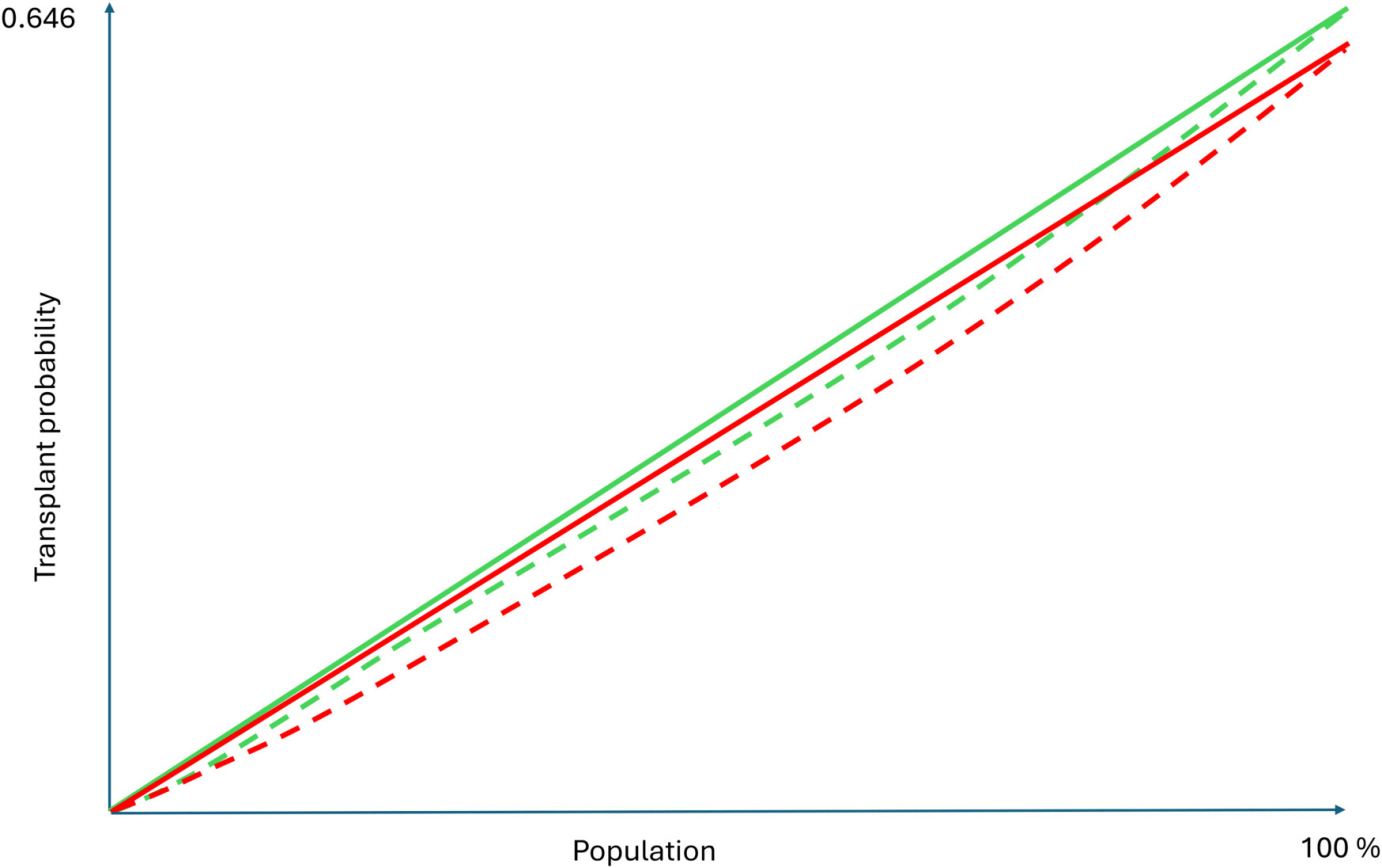
Lorenz curves for Cox based allocation based on maximizing survival and hybrid allocation.

The results reveal that while the equity of access policy has fewer differences between subpopulations, performance is significantly worse for all subpopulations for at least one allocation performance metric. The same also applies to the hybrid policy, although the performance differences are less substantial. Thus, the performance obtained when maximizing survival effectiveness is strictly better for some subpopulations while avoiding negative effects for others. These results are visualized by the green Lorenz curve for the maximizing effectiveness policy and the red Lorenz curve for the hybrid policy in Figure 5. According to the equity definition provided in Subsection 2.2, the survival maximizing solution might thus be equitable. Let us close this case study by noting that maximizing effectiveness is also associated with prioritizing younger and healthier patients and therefore associated with equity of outcomes in relative terms (as mentioned in Subsection 2.1).

## 4 Discussion

In this study we have analyzed the trade-offs between effectiveness, equity, and explainability as a result of predictive and prescriptive analytics to improve the allocation of health resources. Our initial theory-based hypothesis has been that trade-offs between the three aforementioned constructs exist and that demanding more explainability limits the effectiveness and equity attainable.

The analysis is based on two case studies from very different contexts and covering different types of needs and services. The first considers FP services in Uganda (low-income). The second case study addresses the advanced surgical intervention of kidney transplantation in the US (high-income). Based on the results obtained from two very different case studies, the key finding is that the initial hypothesis is partially falsified.

Providing an in depth interpretation of the results from the viewpoint of the initial hypothesis, we firstly notice that the least explainable, “black-box,” models at best perform marginally better in terms of effectiveness and equity in comparison to quite explainable alternatives. In the FP case study, the difference was less than one percent. In the transplant allocation study, the differences were largely non-significant. While this evidence from two very different simulation case studies can be viewed to have limited strength and external validity, it points at the marginal gains that black-box models may achieve over extant models. The case studies thereby exemplify the importance of developing and testing approaches with varying degrees of explainability.

Sacrificing explainability does not necessarily add much strength to the modeling of the relationships in the presented operational model, i.e., the modeled impact of the resources allocated on process measures such as service access and quality and/or on health and well-being outcomes. In the FP case study, these relationships were captured equally well by simple, transparent and explainable analytics. For the transplant case study, one may argue that even the advanced black-box model of random survival forests was unable to improve over the modest survival predictions of the rather explainable Cox proportional hazard and decision tree models. Such phenomena may occur especially when the outcomes can intrinsically only be explained partially by the relations and predictors at hand. Adding less explainable technologies will not resolve the remaining “noise.” As health and well being depend on many factors beyond resource allocation, the two very different case studies can thus be interpreted to illustrate this same fundamental prediction accuracy problem.

This brings us to the importance of evidence when considering predictive and prescriptive analytics for health resource allocation. An important question to be asked when considering less explainable analytics is how much benefit these bring in the two dimensional effectiveness-equity space (that is facing the reader in the Figures 1, 3, 4). The case studies presented evidence from simulation studies. Subsequent experimental evidence can help to assess practical effectiveness-equity performance of black-box technologies. Unfortunately, the evidence base on healthcare analytics has advanced slowly (82).

Strong evidence of effectiveness and equity can also reduce the need for explainability. Health systems and medical professionals have a history of adopting drugs and medical technologies supported by strong evidence, even when the workings and outcomes are not fully explained or understood (54). Accordingly, technology assessment protocols and approval regulations are being adjusted to include embedded AI and analytics (83). Such progress is especially valuable for cases in which “black box” analytics outperform more explainable alternatives.

On the same theme, the FP case study states that prescriptive analytics which planners and professionals perceive to lack explainability is unlikely to be implemented as it is not consistent with their evidence based values. Associations of medical professionals are issuing guidance on adoption of advanced analytics so as to facilitate conduct according to professional standards (e.g., regarding effectiveness and equity) when considering to use these technologies in practice (54, 55). Indeed, this has led to the use of the term “augmented intelligence” as an alternative for “artificial intelligence” to express the view that the technologies augment the intelligence of the human professionals involved, rather than substitute their intelligence (55). It has been argued that “*when there are overarching concerns of justice—that is, concerns about how we should fairly allocate resources—ex ante transparency about how the decisions are made can be particularly important…. we may prefer to trade off some accuracy, the price we pay for procedural fairness*” (60). In view of the results obtained, this can be interpreted to argue against the least explainable analytics for the case studies hand.

On the other hand, the FP case study makes clear that the base case of not using any form of augmentation by prescriptive analytics provided the worst solutions in the effectiveness-equity space. The same can be concluded for the FIFO-based kidney allocation policy, which can be viewed to be explainable and to provide equitable access but is significantly lacking in terms of effectiveness and equity of outcomes. Analytics can learn and replicate resulting biases encoded in available data from non optimal allocation practices, which is especially undesirable when transparency and explainability are limited (20). Hence, the case studies can also be interpreted to illustrate that explainable analytics provides an opportunity to overcome historic effectiveness and equity shortcomings.

**Figure 6:**
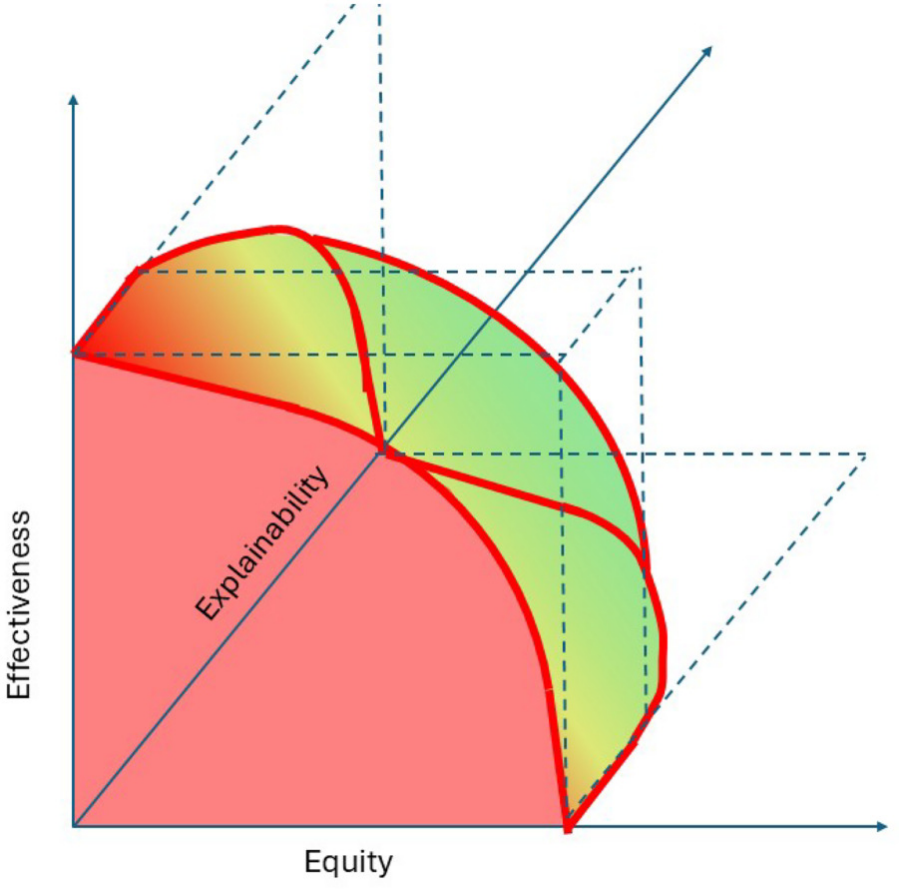
A revised perspective on the trade-offs between effectiveness, equity, and explainability.

The above leads us to an adjusted version of the theoretically developed Figure 1 which adds a practical, evidence-based perspective, as present in Figure 6. It highlights the very limited benefits, if any, of choosing less explainable models beyond a certain explainability threshold. As mentioned, the real world effectiveness may even diminish beyond this point because of implementation challenges. Obviously, the actual shape of Figure 6 may vary across allocation problems and over time as the scientific field of explainable analytics advances. Future empirical studies will be important.

Lastly, let us therefore spend a few words on explainability questions that are especially relevant for future studies on the allocation of scarce resources. What needs to be explainable, the working of the analytics, the outcomes, both, and for whom and by whom? More specifically, one may wonder whether the workings, outcomes, or both should be explainable to all patients? How can we assess whether an explanation is correct and leads to the desired understanding? For instance, who understands the supposedly explainable Cox proportional hazard models and the relationship between deceased censored graft survival and patient outcomes such as life expectancy and well-being? Is it enough to explain the effectiveness and equity of outcomes at a subpopulation level, or is it required to explain *why* a resource allocation is effective and equitable? For instance, is it necessary for patients to understand why the team visits another village and not theirs? Why is it fair and just that a donor kidney is allocated to another patient while my patient is in worse health and waits longer? Does explainability refer to the logic of the model, or to the embedded norms, values and ethics (54, 84)? With many of these questions open, advancement of the explainability of fairness and justice regarding the working of analytics and regarding the equity of outcomes forms a research direction that can accelerate the uptake of advanced analytics in support of SDG3.

### 4.1 Limitations

A first limitation is that the analysis is based on two illustrative case studies. Despite being very different and from both extremes of the low-income high-income continuum, the comparable results obtained from these illustrations may have limited general validity. Hence our call for further empirical research to validate or falsify the findings summarized in Figure 6. This will also serve to address the second abovementioned limitation; both studies simulate allocation policies and hence can be viewed to provide weak evidence. Third, let us repeat that the case studies only cover some applications of non-explainable analytics and that other, future, non explainable models may provide different results. Future research might identify health resource allocation domains for which they attain more substantial performance improvement.

## Data Availability

Publicly available datasets were analyzed in this study. This data can be found here: https://optn.transplant.hrsa.gov/data/view-data-reports/request-data/.
