# Supplementary material for: The effectiveness, equity and explainability of health service resource allocation—with applications in kidney transplantation & family planning
Source: Front Health Serv. 2025 May 15;5:1545864. doi: 10.3389/frhs.2025.1545864 (PMC12119484; doi:10.3389/frhs.2025.1545864)
Supplement: Supplementary file 1 [file Datasheet1.pdf]

Supplementary Table 1: Equity of prediction performance after undersampling. CPH: Cox proportional hazard model, STD: Survival decision tree, RSF: Random survival forest.

**Category**	**Group**	**CPH** μ (σ)	**SDT** μ (σ)	**RSF** μ (σ)
**Calibration**				
Brier Score				
	Overall	0.222 (0.003)	0.226 (0.003)	0.222 (0.003)
	Female	0.216 (0.003)	0.219 (0.004)	0.215 (0.003)
	Male	0.227 (0.005)	0.231 (0.005)	0.225 (0.002)
	Amer Ind/Alaska Native	0.254 (0.023)	0.252 (0.034)	0.247 (0.023)
	Asian	0.199 (0.009)	0.206 (0.011)	0.193 (0.009)
	Black	0.242 (0.005)	0.244 (0.005)	0.238 (0.005)
	Hispanic	0.205 (0.003)	0.209 (0.006)	0.208 (0.004)
	Multi-racial	0.250 (0.073)	0.255 (0.091)	0.272 (0.083)
	Native Hawaiian/Pacific	0.186 (0.021)	0.202 (0.051)	0.184 (0.033)
	White	0.218 (0.005)	0.223 (0.005)	0.217 (0.003)
**Mean Signed Error**				
	Overall	-0.049 (0.011)	-0.052 (0.012)	-0.046 (0.013)
	Female	-0.024 (0.014)	-0.033 (0.016)	-0.023 (0.015)
	Male	-0.065 (0.012)	-0.064 (0.013)	-0.061 (0.014)
	Amer Ind/Alaska Native	-0.106 (0.098)	-0.142 (0.073)	-0.136 (0.106)
	Asian	0.033 (0.035)	0.041 (0.029)	0.059 (0.027)
	Black	-0.085 (0.016)	-0.088 (0.019)	-0.082 (0.021)
	Hispanic	0.004 (0.019)	-0.002 (0.025)	0.000 (0.014)
	Multi-racial	-0.279 (0.197)	-0.199 (0.165)	-0.326 (0.203)
	Native Hawaiian/Pacific	0.095 (0.098)	0.023 (0.133)	0.079 (0.101)
	White	-0.055 (0.013)	-0.056 (0.015)	-0.051 (0.014)
**Discrimination**				
C-Index				
	Overall	0.608 (0.008)	0.595 (0.009)	0.613 (0.007)
	Female	0.579 (0.007)	0.576 (0.014)	0.588 (0.011)
	Male	0.624 (0.010)	0.606 (0.011)	0.627 (0.008)
	Amer Ind/Alaska Native	0.658 (0.034)	0.645 (0.058)	0.669 (0.054)
	Asian	0.628 (0.037)	0.614 (0.035)	0.639 (0.025)
	Black	0.581 (0.014)	0.560 (0.010)	0.573 (0.011)
	Hispanic	0.652 (0.013)	0.625 (0.013)	0.635 (0.022)
	Multi-racial	0.637 (0.204)	0.592 (0.135)	0.530 (0.209)
	Native Hawaiian/Pacific	0.814 (0.063)	0.737 (0.097)	0.747 (0.100)
	White	0.609 (0.011)	0.610 (0.015)	0.635 (0.011)

Supplementary Table 2: Results of the equitable FIFO policy for each three prediction mod-els. TP: transplant probability, WTUT: waiting time until transplant, ESAT: expected survival after transplant.

	**TP**		**WTUT**		**ESAT**	
**Group**	μ	σ	μ	σ	μ	σ
**Cox Proportional Hazard**						
Total Population	0.610	0.019	1253	76	2579	10
Amer Ind/Alaska Native, Non-Hispanic	0.566	0.055	1155	85	2640	129
Asian, Non-Hispanic	0.592	0.036	1276	85	2561	63
Black, Non-Hispanic	0.587	0.022	1263	77	2608	23
Hispanic/Latino	0.626	0.018	1194	77	2561	35
Multiracial, Non-Hispanic	0.648	0.088	1358	142	2650	188
Native Hawaiian/Other Pacific, Non-H	0.614	0.145	1276	133	2487	229
White, Non-Hispanic	0.623	0.022	1267	78	2565	21
**Decision Tree**						
Total Population	0.610	0.019	1253	76	2584	21
Amer Ind/Alaska Native, Non-Hispanic	0.566	0.055	1155	85	2635	254
Asian, Non-Hispanic	0.592	0.036	1276	85	2639	114
Black, Non-Hispanic	0.587	0.022	1263	77	2603	40
Hispanic/Latino	0.626	0.018	1194	77	2607	54
Multiracial, Non-Hispanic	0.648	0.088	1358	142	2575	392
Native Hawaiian/Other Pacific, Non-H	0.614	0.145	1276	133	2671	362
White, Non-Hispanic	0.623	0.022	1267	78	2553	40
**Random Survival Forests**						
Total Population	0.610	0.019	1253	76	2834	90
Amer Ind/Alaska Native, Non-Hispanic	0.566	0.055	1155	85	2861	122
Asian, Non-Hispanic	0.592	0.036	1276	85	2849	95
Black, Non-Hispanic	0.587	0.022	1263	77	2836	89
Hispanic/Latino	0.626	0.018	1194	77	2837	81
Multiracial, Non-Hispanic	0.648	0.088	1358	142	2841	193
Native Hawaiian/Other Pacific, Non-H	0.614	0.145	1276	133	2841	202
White, Non-Hispanic	0.623	0.022	1267	78	2829	100

Supplementary Table 3: Results of the hybrid allocation policy. TP: transplant probability WTUT: waiting time until transplant, ESAT: expected survival after transplant.

	TP		WTUT		ESAT	
**Group**	μ	σ	μ	σ	μ	σ
**Cox Proportional Hazard**						
Total Population	0.623	0.019	988	70	2648	7
Amer Ind/Alaska Native, Non-Hispanic	0.550	0.052	707	184	2643	71
Asian, Non-Hispanic	0.549	0.035	1231	141	2630	38
Black, Non-Hispanic	0.644	0.021	835	62	2671	12
Hispanic/Latino	0.600	0.017	1056	89	2631	17
Multiracial, Non-Hispanic	0.674	0.079	903	152	2615	86
Native Hawaiian/Other Pacific, Non-H	0.548	0.111	1805	480	2651	118
White, Non-Hispanic	0.627	0.024	1064	79	2638	9
**Decision Tree**						
Total Population	0.610	0.019	1253	76	2584	21
Amer Ind/Alaska Native, Non-Hispanic	0.566	0.055	1155	85	2635	254
Asian, Non-Hispanic	0.592	0.036	1276	85	2639	114
Black, Non-Hispanic	0.587	0.022	1263	77	2603	40
Hispanic/Latino	0.626	0.018	1194	77	2607	54
Multiracial, Non-Hispanic	0.648	0.088	1358	142	2575	392
Native Hawaiian/Other Pacific, Non-H	0.614	0.145	1276	133	2671	362
White, Non-Hispanic	0.623	0.022	1267	78	2553	40
**Random Survival Forests**						
Total Population	0.610	0.019	1253	76	2834	90
Amer Ind/Alaska Native, Non-Hispanic	0.566	0.055	1155	85	2861	122
Asian, Non-Hispanic	0.592	0.036	1276	85	2849	95
Black, Non-Hispanic	0.587	0.022	1263	77	2836	89
Hispanic/Latino	0.626	0.018	1194	77	2831	81
Multiracial, Non-Hispanic	0.648	0.088	1358	142	2841	193
Native Hawaiian/Other Pacific, Non-H	0.614	0.145	1276	133	2841	202
White, Non-Hispanic	0.623	0.022	1267	78	2829	100
